# Supplementary material for: Global seroprevalence of SARS-CoV-2 antibodies: A systematic review and meta-analysis
Source: PLoS One. 2021 Jun 23;16(6):e0252617. doi: 10.1371/journal.pone.0252617 (PMC8221784; doi:10.1371/journal.pone.0252617)
Supplement: Attachment — Submitted filename: Review SARS2_SeroReview_PlosOne.pdf [file pone.0252617.s002.pdf]

Review of “*Global seroprevalence of SARS-CoV-2 antibodies: a systematic review and meta-analysis*” for Plos One

January 2021

The manuscript summarises the serology studies available for SARS-CoV-2 through a systematic review and meta-analysis of the resulting data. In addition to describing the overall global seroprevalence, they characterise seroprevalence in various demographic groups e.g. age, gender, country, ethnicity, type of patient/sample etc. Since the approach taken corrects for bias such as lack of sensitivity/sensitivity or demographic adjustments in the original publications the results here should provide an increased accuracy in the estimates.

The manuscript is interesting and from what I understood from the methods, technically sound. However, it would benefit from some clarifications that I detail below:

- The rationale for needing sero-surveys and a review of sero-surveys is not clear. For example, but not exclusively, in the introduction (3rd paragraph), the authors mention that is increasingly important to measure baseline prevalence of antibodies in the vaccine era. While I agree with importance of understanding serological patterns, sero tests are not being done prior to vaccination nor is being taken into account for the number of doses distributed since there is limited supply, and is also not being used for prioritizing groups. So why is it important that we know this?
- Correction of seroprevalence estimates: This needs a bit more explanation and clarification throughout text and tables. For corrected seroprevalences, did you correct all studies or do you use a mixture of published corrections and corrections made in this study? Either way how do you ensure unbiased/equivalent corrections? What sort of independent evaluations did you base your corrections, are these for sensitivity/specificity values provided by commercial kits? What about lab-to-lab variations and in-house assays? What type of sensitivity analysis was conducted on uncorrected data and for what?
  How do you correct for power of the studies? The sample size of studies would have been planned taking into account the population size and demographic of the region, hence providing a powered measure of seroprevalence, but many not.
  How did the meta-analysis account for the level of risk of bias identified? And how can we interpret this risk of bias?

Other comments:

Introduction
2nd paragraph: ‘previous infection’ – infection or exposure?
4th paragraph:
- what gap? There was no clear gap identified up to here.
- what is the start and end dates for the lit review?
- ‘true burden’ – I wonder if this is the best term (which is mentioned throughout the manuscript). Doesn’t burden refer to mortality and morbidity? Or at least something that incurs some sort of cost. Many, if not most, seropositives will have been asymptomatic.

Data sources:
- Is there a reason to exclude PubMed?
- Who is the librarian? At least add the affiliation.
- key eligibility criteria/ search words should be specified in the main text.

Study selection: ‘SARS-CoV-2 infection’ –do you mean studies that included only previously PCR positives?
Associated factors: there are far more studies for high-income countries, how do you take study effort into account for global or even large regional scales?

Results:
- what is considered general and special populations?
- blood donors seem to be considered as general population, given they are typically young and healthier/fiter than average, are they not a special population?
- the time window for these estimates need to be stated at the start of the results. I would imagine that now, seroprevalence is considerably higher in many regions/groups.
Table 4: Could remove rows for reference as this information is already in columns. The risk seems higher for children than adults? This seem to contradict many studies no?
I wonder if some of the tables can be transformed into plots for an easier visualization?
Conclusion:
- 2nd paragraph: Or baseline health…. The sentence starting 'Given' is important and should be expanded. How does Community transmission impact SARS-CoV-2 transmission? It currently read transmission impacts transmission which seems a bit circular and empty. Is community transmission a proxy or behaviour?
- what are the units of (24.0 local vs 11.9 national vs 15.7 regional)?
- the 11.9 ratio values is without applying spatial heterogeneity in under-ascertains both between countries and within a country - and is biased by the countries that had capacity to perform a serological test. How would these estimates change if these heterogeneities were included?
P17 1st parag: 'may not seroconvert' - or antibodies could have wained by the time of blood collection...
Many studies have repeated patients. Was this considered?
P17 2nd parag: 'there may be other factors…' such as what?
P17 3rd parag: given the different level of scrutiny of these types of articles, do you think the results are comparable?
